# The relationship between physical activity and procrastination behavior among Chinese university students: a chain mediated effect of body self-esteem and overall self-esteem

**DOI:** 10.3389/fpubh.2024.1434382

**Published:** 2024-07-09

**Authors:** Sunyoumeng Zhuan, Jinjin Cao, Yongheng Ye, He Li, Qinqin Zhang, Xin Wang

**Affiliations:** ^1^School of Physical Education, Xuzhou University of Technology, Xuzhou, Jiangsu, China; ^2^Department of Joint Surgery and Sports Medicine of Zhuhai People’s Medical College, Zhuhai, Guangdong, China; ^3^Linyi Vocational College of Science and Technology, Linyi, Shandong, China; ^4^Zhuhai Clinical Medical College of Jinan University, Zhuhai, Guangdong, China; ^5^Nanxishan Hospital of Guangxi Zhuang Autonomous Region, Guilin, Guangxi, China; ^6^Departments of Thyroid and Bread Surveillance, Nanjing Hospital of Guangzhou Autonomous Region, Guilin, Guangxi, China; ^7^School of Physical Education, Zhengzhou University, Zhengzhou, Henan, China

**Keywords:** body self-esteem, overall self-esteem, procrastination behavior, serial mediation effect, physical activity

## Abstract

**Objective:**

This study aimed to explore the relationship between physical activity and procrastination behavior among college students and the serial mediating roles of body self-esteem and overall self-esteem.

**Methods:**

A cross-sectional survey was conducted involving 1,018 college students. The Physical Activity Rating Scale, the College Students’ Physical Self-perception Profile, the Self-Esteem Scale, and the Chinese Procrastination Scale were utilized. Serial mediating effect analysis and Bootstrap analysis were applied to the data.

**Results:**

(1) Individuals engaging in high levels of physical activity demonstrated significantly lower levels of procrastination behavior compared to those with moderate or low levels of physical activity (Partial η^2^ = 0.01, *F* = 2.762, *p* < 0.01). (2) Physical activity was positively correlated with body self-esteem (*r* = 0.37, *p* < 0.01) and overall self-esteem (*r* = 0.29, *p* < 0.01), and negatively correlated with procrastination behavior (*r* = −0.14, *p* < 0.01). body self-esteem was positively correlated with overall self-esteem (*r* = 0.11, *p* < 0.01) and negatively correlated with procrastination behavior (*r* = −0.13, *p* < 0.01). Overall self-esteem also showed a significant negative correlation with procrastination behavior (*r* = −0.26, *p* < 0.01). (3) Mediation effect analysis indicated that body self-esteem and overall self-esteem played partial mediating roles in the relationship between physical activity and procrastination behavior among college students. The mediating effects were composed of three pathways: “Physical Activity → body self-esteem → Procrastination Behavior,” “Physical Activity → Overall Self-Esteem → Procrastination Behavior,” and “Physical Activity → body self-esteem → Overall Self-Esteem → Procrastination Behavior,” with the effect sizes accounting for 27.70, 12.26, and 7.28% of the total effect, respectively.

**Conclusion:**

The findings of this study lead to several key conclusions regarding the relationship between physical activity, self-esteem, and procrastination behavior among college students. Firstly, engagement in moderate levels of physical activity is more effective in reducing procrastination behavior among college students. Secondly, participation in high volumes of physical activity is associated with increased levels of both body self-esteem and overall self-esteem. Lastly, physical activity not only directly impacts procrastination behavior but also indirectly influences it by enhancing body self-esteem and overall self-esteem, thereby further reducing the individual’s level of procrastination. These conclusions suggest that physical activity plays a multifaceted role in mitigating procrastination behavior in college students. By promoting self-esteem, physical activity can foster a positive psychological state that is conducive to proactive behavior and academic success. The study’s findings underscore the importance of integrating physical activity into college students’ daily routines as a strategy to combat procrastination and enhance self-esteem.

## 1 Introduction

### 1.1 The concept and severity of procrastination behavior

Procrastination, characterized as a negative behavioral or coping mechanism, is defined as the voluntary postponement of task initiation despite awareness of potential adverse consequences, and it is a prevalent behavioral tendency across various domains with temporal and contextual stability (1). Steel (1) posits that procrastination stems from a failure in self-regulation, with its main features including voluntariness, avoidance, and irrationality. Pang Weiguo (2) found that nearly 40% of students reported that their procrastination behavior had a detrimental effect on academic performance. A cross-sectional study by Kim et al. (3) among college students revealed an increasing trend in the prevalence of procrastination issues. Procrastination has numerous adverse effects on the college student population. Research has shown that procrastination not only diminishes work performance and academic achievement but may also lead to feelings of regret, shame, and adverse emotions such as depression and anxiety (4, 5). It may result in increased stress levels, decreased academic grades, test anxiety, social anxiety, reduced self-efficacy, lowered self-esteem, and even negative impacts on physical health (6). Therefore, it is imperative to explore the mechanisms underlying procrastination behavior among college students and to seek effective measures for its prevention and control in higher education settings.

### 1.2 Physical activity and procrastination behavior based on the theory of planned behavior

The Theory of Planned Behavior (TPB) is one of the most successful theoretical models for explaining and predicting physical exercise, which can assist researchers in clarifying the impact process mechanism of physical exercise on academic procrastination (7). TPB focuses on the relationship between intentions and behavior, offering explanations and predictions for individual actions. A core proposition of the theory is that behavioral intentions can directly determine behavior, with behavioral attitudes, subjective norms, and perceived behavioral control being the three predictive factors that influence the strength of behavioral intentions. Consequently, the more positive an individual’s attitude toward the target, the stronger the subjective norms, and the greater the perceived behavioral control, the stronger the intention to perform target-oriented behavior, and the more likely the individual is to engage in the behavior. Empirical studies have shown that TPB can account for nearly 40% of the variance in physical exercise behavior, and all predictive factors of TPB can significantly predict the state of college students’ participation in physical exercise (8). TPB is also one of the more frequently used theories in procrastination behavior research. Lin (9) used TPB to explore the procrastination behavior of college students and further applied the TPB framework to conduct intervention studies on procrastination behavior among college students, confirming that TPB can negatively predict procrastination behavior. Through literature review, it has been found that physical exercise and academic procrastination, as outcome variables of TPB, are both influenced by the factors of the theory, indicating that the two are not isolated individual behaviors and may have an interactive relationship.

Procrastination behavior has increasingly gained the attention of scholars in various disciplines, including education and psychology, with numerous researchers examining the relationship between physical activity and procrastination. The concept of employing exercise to counteract procrastination was initially proposed by American Psychologists Burka and Yuen in their co-authored book “Procrastination: Why You Do It, What to Do About It Now” (10, 11). They posit that engaging in physical exercise can maintain an individual’s physical well-being and enhance their courage to confront tasks that have been postponed, thereby serving as an effective strategy to reduce procrastinatory behavior. In a 2012 review on intervention methods for procrastination, Chinese scholar Xu Jinfeng also identified physical exercise as a form of behavioral intervention. Drawing on Time Motivation Theory (TMT) and self-regulation theory, Xu and others emphasize the importance of time management and self-discipline in overcoming procrastinatory habits. They argue that activities such as physical exercise, military training, and social practice are effective means to strengthen self-discipline and enhance time management skills. Many studies on Chinese college students have already utilized longitudinal data. These data indicate that physical exercise and issues like anxiety and depression among college students are processes of change, and there is heterogeneity among different groups (12, 13). Yiming Tao et al. (14) conducted an empirical study involving 610 college students to explore the relationship between procrastination and physical activity. The results revealed a significant negative correlation between the two, indicating that higher levels of physical activity are associated with lower levels of procrastination behavior. In another empirical study, Kai Ren et al. (15) surveyed 687 Chinese college students using a cross-sectional approach to investigate the impact of physical activity levels on academic procrastination. After conducting correlation and regression analyses, they found that physical activity levels have a significant negative impact on academic procrastination among college students. Furthermore, their study suggests that physical activity levels are an effective intervention measure for directly and indirectly reducing procrastination behavior in college students. Based on these theoretical underpinnings, the use of physical exercise to correct individual procrastination behavior is theoretically plausible. Empirical evidence highlighting the significance of regular physical activity in the context of procrastination has begun to emerge, supporting the initiation of studies in this area. Consequently, this study hypothesizes a significant negative correlation between physical activity and procrastination behavior among college students (H1).

### 1.3 Body self-esteem and procrastination behavior

body self-esteem, a concept closely intertwined with social evaluation, pertains to an individual’s level of satisfaction or dissatisfaction with various aspects of their body (16). This form of self-esteem is not only about personal perceptions of one’s physical form, appearance, and functionality but also significantly relates to self-evaluation in social interactions. Body self-esteem plays a crucial role in maintaining mental health and enhancing life satisfaction. Within the academic context, bolstering students’ body self-esteem is an integral component of university mental health promotion initiatives. As a multidimensional assessment indicator, body self-esteem encompasses not only cognition of one’s physical condition but also self-assessment of personal physical attractiveness and physical efficacy. Moreover, there is a close association between body self-esteem and the extent of an individual’s engagement in physical activities (17). Active participation in sports can improve physical fitness and (18), through a sense of enhanced physical capability and achievement in sports, elevate body self-esteem (19–21). This enhancement in positive self-perception and self-worth can encourage proactive behavior in other life domains, including academic success, social skills, and personal development. Therefore, higher education institutions should prioritize the promotion of students’ body self-esteem through physical education and engagement in physical activities. Designing and implementing a diverse range of physical activity programs can cater to the varying interests and needs of students, helping to establish a positive self-image and strengthen self-efficacy, thereby fostering significant advancements in mental health and personal growth. Some studies have confirmed the significant positive predictive effect of physical exercise on body self-esteem, indicating that regular physical training can elevate levels of body self-esteem, with higher levels of physical activity correlating with higher levels of body self-esteem (22, 23). Furthermore, empirical research by Ren et al. (24) has shown a significant negative correlation between body self-esteem and procrastination behavior. Based on these findings, this study hypothesizes that body self-esteem mediates the impact of physical activity on college students’ procrastination behavior (H2).

### 1.4 Overall self-esteem and procrastination behavior

Global self-esteem, which reflects an individual’s perception of their self-worth and self-acceptance, is a comprehensive and enduring sense of self and self-evaluation (25). Zamani et al. (26) discovered that physical activity positively predicts levels of self-esteem. Corresponding research has also demonstrated that engagement in sports activities can robustly enhance overall self-esteem (22). Concurrently, the exploration of the psychological underpinnings of procrastination behavior has emerged as a focal point within the academic community. A substantial body of research indicates a negative correlation between self-esteem and procrastination, with individuals exhibiting lower self-esteem being more prone to procrastination, while those with higher self-esteem are less likely to procrastinate (27, 28). In light of these findings, this study hypothesizes that overall self-esteem serves a mediating role in the influence of physical activity on procrastination behavior among college students (H3).

### 1.5 Overall self-esteem and body self-esteem

According to the multidimensional and hierarchical self-esteem structure model proposed by Shavelson et al. (29), body self-esteem, as a critical sub-dimension of overall self-esteem, can significantly influence an individual’s global self-esteem. Specifically, fluctuations in body self-esteem at a lower hierarchical level can correspond to changes in overall self-esteem. Empirical research supports this notion, showing that engagement in physical activity is associated with an enhancement of both body self-esteem and overall self-esteem (30). Furthermore, studies focusing on the college student population have confirmed the close link between body self-esteem and overall self-esteem, demonstrating a significant positive correlation between the two, with body self-esteem having a substantial positive impact on overall self-esteem (17). These findings lead to the conclusion that body self-esteem is not only a fundamental component of the self-esteem structure but also plays a pivotal role in the development and maintenance of self-esteem. The improvement of body self-esteem can augment an individual’s sense of self-worth and self-efficacy, as well as their confidence and satisfaction in social interactions. Zamani et al. (26) found that body self-esteem acts as an intermediary in the pathway between levels of physical activity and overall self-esteem. Consequently, higher education institutions should emphasize the enhancement of students’ body self-esteem through physical activities and exercise as part of their mental health education and promotion efforts, thereby fostering the healthy development of overall self-esteem. This approach can effectively assist students in establishing a more positive and comprehensive self-concept, laying a solid foundation for their future academic achievements, career development, and social adaptation. Based on these considerations, this study hypothesizes that body self-esteem and overall self-esteem exert a serial mediating effect on the impact of physical activity on college students’ procrastination behavior (H4).

### 1.6 Research purpose

In summary, this study aims to investigate the differences in body self-esteem, overall self-esteem, and procrastination behavior levels among college students with varying levels of physical activity engagement. By analyzing these differences, the study seeks to verify the correlations between physical activity, body self-esteem, overall self-esteem, and procrastination behavior. Subsequently, the research aims to further examine the potential mechanisms by which body self-esteem and overall self-esteem mediate the impact of physical activity on procrastination behavior among college students. The ultimate goal is to provide a theoretical foundation for the intervention and correction of procrastination behavior in the college student population.

## 2 Research subjects and methods

### 2.1 Research subjects

**Figure 1:**
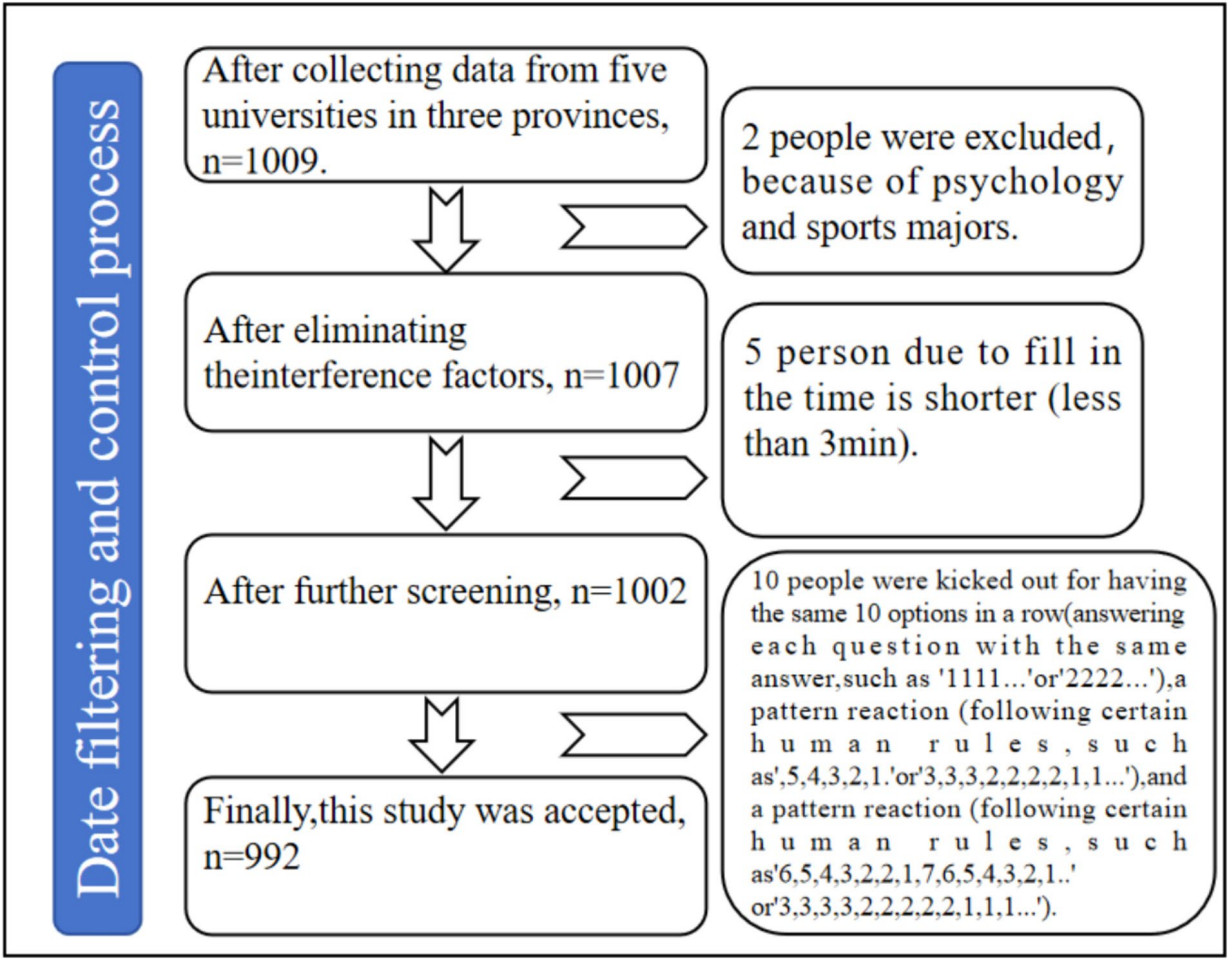
The screening process.

This study, conducted between January and February 2024, employed a cluster random sampling method, selecting 1,000 college students from a university in Henan Province as the research subjects. A total of 1,009 questionnaires were distributed. After collection, questionnaires with invalid responses, those completed in an unreasonably short time (less than 200 s), with more than 10 consecutive identical choices, or with high homogeneity were excluded, the flow chart of screening invalid questionnaires is shown in Figure 1. This process yielded 992 valid questionnaires, resulting in a response rate of 98.32%. The sample comprised 519 males (52.32%) and 473 females (47.68%), with participant ages ranging from 18 to 23 years old (mean age: 20.35 ± 3.98 years).

In the present study, we employed a cluster random sampling method to select the research subjects. Specifically, we initially categorized the student population into distinct “clusters” based on the academic departmental structure of the university. This clustering approach was predicated on the commonalities among students within academic departments regarding educational background, learning environment, and campus culture, thereby aiming to enhance the homogeneity and comparability of the sample. Within each academic department, we further stratified the sample according to the students’ year of study and major to ensure diversity and representativeness. Subsequently, we utilized a simple random sampling technique to select a predetermined number of students from each cluster as the subjects of our survey, thereby augmenting the randomness and representativeness of the sample. The survey was conducted in a unified group setting, utilizing an online questionnaire platform (Questionnaire Star) to administer the survey to the students. The principal investigators, including the researcher and trained educators, adhered to the principles of the Declaration of Helsinki and obtained ethical approval from the Ethics Committee of the School of Basic Medical Sciences at Zhengzhou University. Approval from the school’s educational administration department and the cooperation of the class teachers were secured. During the students’ self-study time (approximately 15 min), the purpose of the study was explained, and the principles of voluntary participation, anonymity, and honest response were emphasized. Subsequently, the students completed the online survey by scanning a QR code in a quiet classroom setting using their smartphones.

### 2.2 Research methods

#### 2.2.1 Physical activity rating scale (PARS-3)

The Physical Activity Rating Scale (PARS-3), developed by Hashimoto Kimio and revised by Liang Deqing (31), consists of three dimensions: activity intensity, duration, and frequency. Each dimension contains one item, rated on a 5-point Likert scale, with higher scores indicating greater physical activity levels. Example item: “How many times per week do you engage in moderate-intensity physical activities, such as brisk walking or jogging?” Response options might include: “0 times,” “1–2 times,” “3–4 times,” “More than 5 times.” The scoring method is calculated as “Intensity × (Duration - 1) × Frequency = Total Physical Activity Score,” where scores ≤19 indicate low physical activity, 20–42 indicate moderate physical activity, and ≥ 43 indicate high physical activity. In this study, the total Cronbach’s α for the scale was 0.79. The confirmatory factor analysis (CFA) yielded indices indicative of an acceptable model fit: the normed chi-square statistic (χ^2^/df) was 4.11, the comparative fit index (CFI) reached 0.97, the goodness of fit index (GFI) was 0.96, the Tucker-Lewis index (TLI) stood at 0.95, the root mean square error of approximation (RMSEA) was 0.07, and the standardized root mean square residual (SRMR) measured 0.037. Adhering to the criteria proposed by Schumacker and Lomax (32), these values suggest that the scale is appropriately calibrated for the population under investigation.

#### 2.2.2 Physical self-perception profile for college students

The Chinese version of the PSPP, revised by Xu Xia et al. (33), was utilized. It includes one main scale, body self-worth, and four subscales: sports competence, physical condition, physical attractiveness, and physical strength, comprising a total of 30 items (including 16 reverse-scored items). Example item: “How do you rate your performance in physical activities?” (Response options range from: Very Poor, Poor, Average, Good, to Very Good). The scale uses a converted 5-point Likert scale, with higher scores indicating higher levels of body self-esteem. The overall Cronbach’s α for this scale in the present study was 0.90. The CFA yielded indices indicative of an acceptable model fit: χ^2^/df = 2.91, CFI = 0.96, GFI = 0.95, TLI = 0.95, RMSEA = 0.05, SRMR = 0.04. Adhering to the criteria proposed by Schumacker and Lomax (32), these values suggest that the scale is appropriately calibrated for the population under investigation.

#### 2.2.3 Rosenberg self-esteem scale

The SES, developed by Rosenberg in 1965 and translated by Ji Yifu and Yu Xin (34), is widely used in China. It contains 10 items, with items 1, 2, 4, 6, 7, and 8 being positively scored, and the rest being reverse-scored. The scale uses a converted 5-point Likert scale, with a score range of 10–50 points, where higher scores indicate higher levels of self-esteem. Example item: “I feel that I have a number of good qualities.” (Response options range from: Strongly Disagree, Disagree, Neutral, Agree, to Strongly Agree). The total Cronbach’s α for this scale in this study was 0.92. The CFA yielded indices indicative of an acceptable model fit: χ^2^/df = 3.01, CFI = 0.99, GFI = 0.97, TLI = 0.97, RMSEA = 0.06, SRMR = 0.05. Adhering to the criteria proposed by Schumacker and Lomax (32), these values suggest that the scale is appropriately calibrated for the population under investigation.

#### 2.2.4 General procrastination scale

Focusing on procrastination behavior in learning and daily life contexts among college students, this study employed the General Procrastination Scale developed by Lay (35). The scale, revised by Bao Cuiqiu, consists of 20 items, including 9 reverse-scored items, with all items summed for a total score. It measures individual procrastination behavior using a 5-point Likert scale, where higher scores indicate more severe procrastination. The use of this scale helps to understand the characteristics of procrastination behavior and its manifestations in different life scenarios among college students. Example item: “I often put off starting my homework until the last minute.” (Response options range from: Never, Rarely, Sometimes, Often, to Always). The total Cronbach’s α for this scale in this study was 0.82. The CFA yielded indices indicative of an acceptable model fit: χ^2^/df = 2.92, CFI = 0.96, GFI = 0.95, TLI = 0.95, RMSEA = 0.05, SRMR = 0.05. Adhering to the criteria proposed by Schumacker and Lomax (32), these values suggest that the scale is appropriately calibrated for the population under investigation.

### 2.3 Statistical analysis

The data were analyzed using SPSS 21.0 for normality testing of body self-esteem, overall self-esteem, physical activity, and procrastination behavior, followed by descriptive statistics, reliability testing, and correlation analysis. One-way ANOVA and correlation analysis were conducted using SPSS, while structural equation modeling and fitting of the serial mediation effect model were performed using Amos 24.0 software. After standardizing the research variables, the PROCESS macro’s Model 6 within SPSS was utilized to test the serial mediation effects, specifically examining the mediating roles of body self-esteem and overall self-esteem between physical activity and procrastination behavior (36–38).

## 3 Results

### 3.1 Common method bias test

Data for this study were collected via an online self-report questionnaire through the platform “Questionnaire Star,” which could potentially introduce common method bias. To address this, Harman’s single-factor test was employed to analyze all items involved in the study. The exploratory factor analysis yielded 11 factors with eigenvalues greater than 1, with the first factor accounting for 23.71% of the variance, well below the critical threshold of 40%. This indicates that common method bias did not significantly affect the data in this study.

### 3.2 Differences in body self-esteem, overall self-esteem, and procrastination behavior among various levels of physical activity

A one-way ANOVA was conducted with the amount of physical activity (low, medium, high) as the independent variable and body self-esteem, overall self-esteem, and procrastination behavior as the dependent variables. The results (see Table 1) revealed significant differences in levels of procrastination behavior across the three groups of physical activity levels. Post-hoc tests indicated that individuals engaging in high levels of physical activity demonstrated significantly lower levels of procrastination behavior compared to those with medium and low levels of physical activity (Partial η^2^ = 0.05, *F* = 2.762, *p* < 0.01).

**Table 1:** Analysis of variance (ANOVA) for the differences in variables by different levels of physical activity (M ± SD).

Physical activity level	n	Procrastination behavior
Low	350	3.68 ± 0.905
Medium	328	3.31 ± 0.959
High	314	2.77 ± 1.066
*F*		5.261**
*partial η^2^*		0.05

*p < 0.05, **p < 0.01.

### 3.3 Correlation analysis

Among College Students’ Physical Activity, Physical and Overall Self-Esteem, and Procrastination Behavior Pearson correlation analysis was conducted to examine the relationships among the variables. The results, as presented in Table 2, indicate that physical activity is significantly and positively correlated with both body self-esteem (*r* = 0.37, *p* < 0.01) and overall self-esteem (*r* = 0.29, *p* < 0.01), while it is significantly and negatively correlated with procrastination behavior (*r* = −0.14, *p* < 0.01). Additionally, body self-esteem is significantly and positively correlated with overall self-esteem (*r* = 0.11, *p* < 0.01) and significantly and negatively correlated with procrastination behavior (*r* = −0.13, *p* < 0.01). Furthermore, overall self-esteem is significantly and negatively correlated with procrastination behavior (*r* = −0.26, *p* < 0.01). The significant correlations among the primary variables suggest that further mediation effect analysis is warranted.

**Table 2:** Descriptive statistics and correlation analysis results for each variable.

Physical exercise	M ± SD	1	2	3	4
body self-esteem	3.07 ± 0.92	1.00			
body self-esteem	3.19 ± 0.36	0.37**	1.00		
Overall Self-esteem	3.27 ± 0.53	0.29**	0.11**	1.00	
Procrastination Behavior	3.46 ± 0.954	−0.14**	−0.13**	−0.26**	1.00

*p < 0.05, **p < 0.01.

### 3.4 Serial mediation analysis of physical activity and procrastination behavior

This study utilized Amos 24.0 for structural equation modeling to assess the mediating effects of body self-esteem and overall self-esteem between physical activity and procrastination behavior among college students. The model considered physical activity as the independent variable, body self-esteem and overall self-esteem as mediators, and procrastination behavior as the dependent variable. The analysis revealed that physical activity positively predicted body self-esteem (*β* = 0.34, *p* < 0.01) and overall self-esteem (*β* = 0.22, *p* < 0.01), while it negatively predicted procrastination behavior (*β* = −0.09, *p* < 0.01). Additionally, body self-esteem positively predicted overall self-esteem (*β* = 0.29, *p* < 0.01) and negatively predicted procrastination behavior (*β* = −0.21, *p* < 0.05), and overall self-esteem negatively predicted procrastination behavior (*β* = −0.17, *p* < 0.01; see Table 3).

**Table 3:** Regression analysis of the serial mediation model of body self-esteem and overall self-esteem.

Variable	Equation 1: (Dependent variable: body self-esteem)			Equation 2: (Dependent variable: overall self-esteem)			Equation 3: (Dependent variable: procrastination behavior)		
	β	SE	*t*	β	SE	*t*	β	SE	*t*
Physical activity	0.18	0.01	10.02**	0.19	0.01	3.91**	−0.15	0.02	−1.72**
Body Self-esteem				0.57	0.03	7.25**	−0.22	0.05	−5.46*
Overall Self-esteem							−0.39	0.07	−8.423**
R2	0.21			0.33			0.74		
F	18.39**			29.68**			84.41**		

*p < 0.05, **p < 0.01.

**Figure 2:**
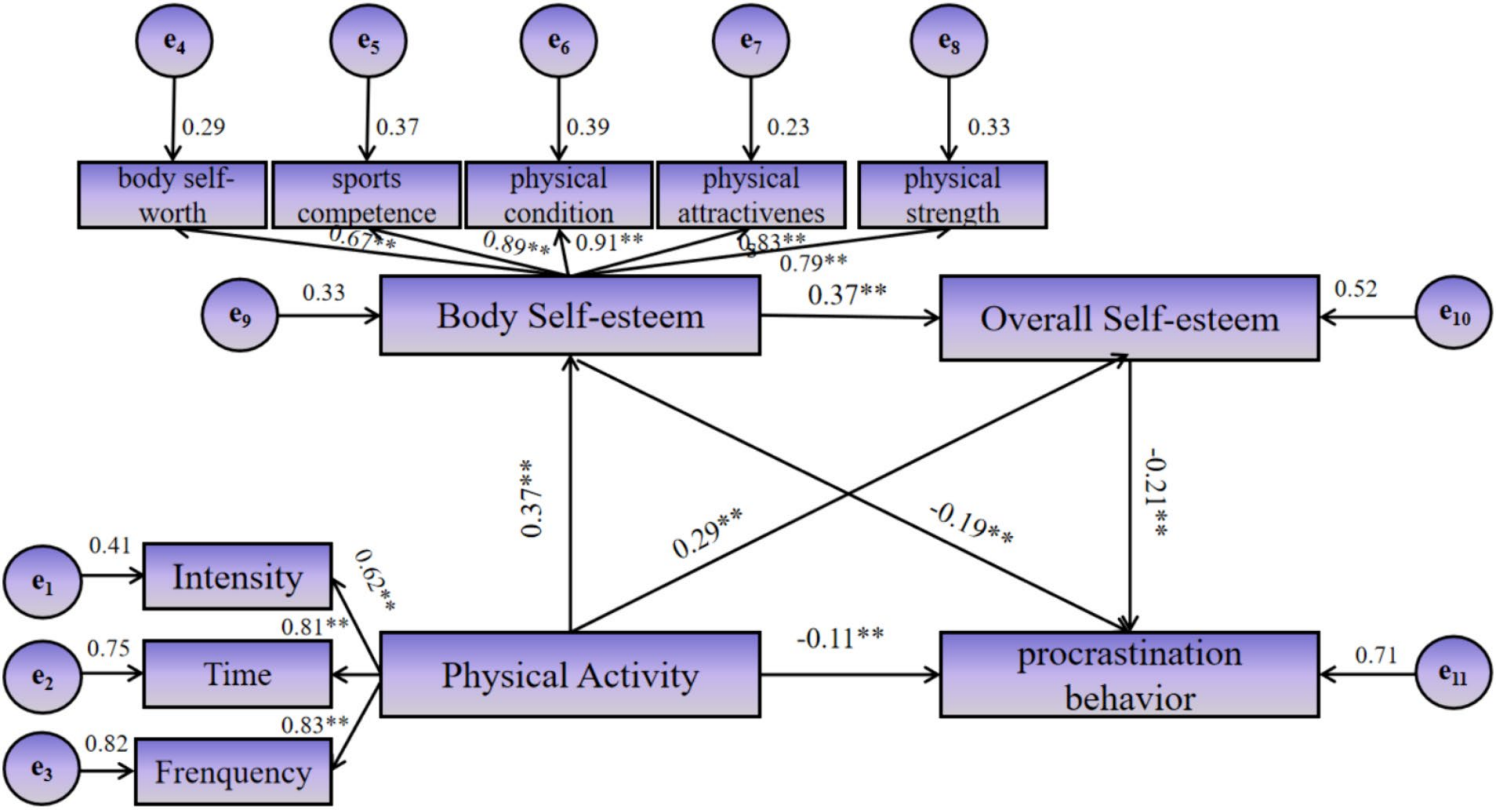
Chain mediated model of body self-esteem and overall self-esteem between physical activity and procrastination behavior.

This study employed the Bootstrap resampling procedure to test the mediation effects of the indirect pathways and to estimate their confidence intervals. The analysis involved 5,000 resamples, and the results are presented in Table 4. The 95% confidence intervals for all three indirect pathways through body self-esteem and overall self-esteem as mediators did not include zero, indicating that the mediation effects were statistically significant. This confirms that both body self-esteem and overall self-esteem significantly mediate the relationship between physical activity and procrastination behavior among college students. The total standardized indirect effect size was −0.122, comprising indirect effects from three distinct mediation pathways. The individual indirect effects were as follows: (1) The “Physical Activity → body self-esteem → Procrastination Behavior” pathway had an effect size of −0.071, accounting for 27.20% of the total effect; (2) The “Physical Activity → Overall Self-Esteem → Procrastination Behavior” pathway had an effect size of −0.032, representing 12.26% of the total effect; (3) The “Physical Activity → body self-esteem → Overall Self-Esteem → Procrastination Behavior” pathway had an effect size of −0.019, constituting 7.28% of the total effect. Additionally, the direct effect of physical activity on procrastination behavior was −0.09, which constituted 43.51% of the total effect. The specific structural equation model is depicted in Figure 2.

**Table 4:** Bootstrap analysis of significance testing for mediating effects.

Effect type	Path	Effect size	BootSE	Boot95%CI		Proportion(%)
				LLCI	ULCI	
Direct effect	Physical Activity → Procrastination Behavior	−0.139	0.036	−0.147	−0.133	53.26%
Indirect effect	Physical Activity → body self-esteem → Procrastination Behavior	−0.071	0.016	−0.077	−0.065	27.20%
	Physical Activity → Overall Self-esteem → Procrastination Behavior	−0.032	0.013	−0.038	−0.026	12.26%
	Physical Activity → body self-esteem → Procrastination Behavior	−0.019	0.001	−0.025	−0.013	7.28%
Total indirect effect	“Physical Activity → body self-esteem → Procrastination Behavior” + “Physical Activity → Overall Self-esteem → Procrastination Behavior” + “Physical Activity → body self-esteem → Overall Self-esteem → Procrastination Behavior”	−0.122	0.016	−0.179	−0.075	46.74%
Total effect	Total Indirect Effect + Direct Effect	−0.261	0.371	−0.359	−0.123	100%

## 4 Discussion

The present study, focusing on the Chinese college student population, examined the relationship between physical activity and procrastination behavior, as well as the serial mediating effects of body self-esteem and overall self-esteem. The specific findings are as follows:

### 4.1 Explanation of sample background

Our study sample comprised university students from an institution in Henan Province, aged between 18 and 23 years. This age cohort is navigating a pivotal phase of higher education, characterized by multifaceted challenges including academic responsibilities, career planning, and personal development. As a distinct demographic, college students may exhibit notable divergences in lifestyle habits, time management, and self-regulatory skills when juxtaposed with high school students or employed adults (39). For instance, the college student population typically enjoys greater autonomy in managing their academic and personal schedules, potentially influencing the frequency and types of physical activities they undertake (40). Furthermore, their on-campus residence often affords increased access to sports facilities and events, which could encourage engagement in physical activities and subsequently impact the dependent variable of our study—procrastination behavior. In deliberating on our findings, we have also contemplated the potential disparities in comparison with studies conducted within different contexts. Relative to high school students (41), college students may confront heightened expectations for independent learning and self-directed management, leading to variations in procrastination patterns. When compared with employed adults, college students might experience less occupational stress but may need to navigate the balance between academic commitments and part-time employment, which could also exert an influence on their procrastinatory tendencies (42).

### 4.2 Direct relationship between physical activity and college students’ procrastination behavior

Empirical analysis from this study revealed a direct link between physical activity and procrastination behavior among college students. It was found that physical activity significantly and negatively predicts procrastination behavior in college students, thus confirming the research hypothesis H1. This finding aligns with the conclusions of the majority of current academic researchers. A study by Li et al. (43) investigated the relationship between physical activity (duration, intensity, and frequency) and procrastination behavior in 564 Chinese college students, yielding similar results. The authors discovered a significant correlation between procrastination and the duration and intensity of physical activity. Additionally, a study by Tao et al. (14), which included 610 college students, explored the relationship between procrastination and physical activity, finding a significant negative correlation. This indicates that higher levels of physical activity are associated with lower levels of procrastination behavior. Consequently, physical activity can be considered an indispensable choice for college students to reduce procrastination behavior, serving not only as a direct and convenient intervention method but also as an effective strategy. Synthesizing the aforementioned analyses, we can conclude that there is a significant negative correlation between college students’ physical activity and procrastination behavior. Participation in physical activity can significantly improve procrastination behavior, providing support for their psychological health and academic achievement.

### 4.3 Mediating role of physical and overall self-esteem in the relationship between physical activity and college students’ procrastination behavior

Additionally, this study discovered that both body self-esteem and overall self-esteem serve as partial mediators in the influence of physical activity on procrastination behavior among college students. The mediating effects accounted for 27.70 and 12.26% of the total effect, respectively, thus supporting hypotheses H2 and H3. This also indicates that physical exercise can indirectly reduce or alleviate procrastination behavior in college students by enhancing their physical and overall self-esteem. Consistent with previous research (22, 44), physical activity was found to be significantly and positively correlated with both body self-esteem and overall self-esteem, suggesting that physical activity can foster body self-esteem in college students, and that an increase in body self-esteem can, in turn, promote overall self-esteem. The model proposed by Sonstroem et al. (45) further elucidates the mechanism by which physical activity influences physical and overall self-esteem. According to this model, an individual’s overall self-esteem is composed of self-concepts from various subdomains, such as the physical, cognitive, and social domains, with specific domains at the lowest level of the model constituting each subdomain. An individual’s overall self-esteem is enhanced as the self-esteem in these lower-level domains increases, and vice versa (46).

Furthermore, the present study found significant negative correlations between both body self-esteem and overall self-esteem with procrastination behavior. The relationship between overall self-esteem and procrastination behavior has been widely studied, and the results of this study are in line with previous findings (28). Direct explorations of the relationship between body self-esteem and procrastination behavior are less common in the literature, with most discussions being framed within the context of overall self-esteem and based on empirical research on the relationship between self-esteem and procrastination behavior, which also aligns with the results of the current study (47).

### 4.4 Serial mediating role of physical and general self-esteem in the relationship between physical activity and procrastination behavior among college students

A significant discovery of this study is the pronounced serial mediating effect of body self-esteem and overall self-esteem on the impact of physical activity on procrastination behavior among college students, which accounts for 7.28% of the total indirect effect, confirming Hypothesis H4. This supports the notion that body self-esteem and overall self-esteem have a protective effect against procrastination behavior. Furthermore, it indicates that physical activity can inhibit the level of procrastination behavior in college students by enhancing body self-esteem and subsequently raising their overall self-esteem. The influence of body self-esteem on procrastination behavior is also mediated by overall self-esteem, suggesting that an increase in body self-esteem can lead to a reduction in procrastination behavior, potentially through the process of elevating an individual’s overall self-esteem. Therefore, the more college students engage in physical activity, the higher their levels of body self-esteem, which in turn boosts their overall self-esteem. Regular physical activity can thus gradually foster a psychologically positive cycle that can contribute to the improvement of procrastinatory symptoms. This highlights the crucial role of body self-esteem and overall self-esteem in the context of physical activity and procrastination behavior among college students.

### 4.5 Limitations

This study has several limitations: (1) The cross-sectional design of this study inherently limits the ability to establish causality. Future research suggests a longitudinal approach to elucidate the time series of the observed relationships. (2) In this study, measures that rely on self-reporting are susceptible to biases derived from individual perceptions, emotions, attitudes, and expectations. Follow-up studies should consider improving the methods used to assess physical activity and more rigorously confirm the findings. (3) The study’s focus on Chinese college students may limit the generalizability of the results across different populations or cultural contexts. It is suggested that the use of different samples in future replication studies can improve the applicability of the study. (4) This study lacks an intervention component and is limited to analyzing the relationship between variables without assessing the effectiveness of the intervention. Future research will incorporate interventions to assess the effectiveness of physical activity in alleviating procrastination behaviors, which will yield more practical insights.

### 4.6 Advantages

The findings support and extend the perspective on the preventive and control measures of procrastination behavior through physical activity, providing theoretical and practical guidance for the prevention and control of procrastination behavior among college students. Current research on procrastination behavior lacks effective intervention measures and treatment methods. The study suggests that by cultivating regular physical activity habits, which enhance body self-esteem and overall self-esteem, the serial mediating effects can activate individual initiative, proactively improve executive function, and reduce the occurrence of procrastination behavior.

## 5 Conclusion

The findings of this study lead to several key conclusions regarding the relationship between physical activity, self-esteem, and procrastination behavior among college students. Firstly, engagement in moderate levels of physical activity is more effective in reducing procrastination behavior among college students. Secondly, participation in high volumes of physical activity is associated with increased levels of both body self-esteem and overall self-esteem. Lastly, physical activity not only directly impacts procrastination behavior but also indirectly influences it by enhancing body self-esteem and overall self-esteem, thereby further reducing the individual’s level of procrastination. These conclusions suggest that physical activity plays a multifaceted role in mitigating procrastination behavior in college students. By promoting self-esteem, physical activity can foster a positive psychological state that is conducive to proactive behavior and academic success. The study’s findings underscore the importance of integrating physical activity into college students’ daily routines as a strategy to combat procrastination and enhance self-esteem.

This study of numerous applications of AI assistance and mental health makes the following recommendations: (1) Application of Artificial Intelligence-Assisted Tools: The research concludes that physical activity plays a significant role in reducing procrastination among college students. Building on this, further research could explore how artificial intelligence (AI) tools can be integrated with physical activities to enhance students’ motivation and consistency in engaging in sports. For instance, developing smart exercise tracking apps that offer personalized workout plans and motivational feedback. (2) Mental Health Monitoring and Intervention: Given the connection between physical activity and psychological well-being, further research should investigate how AI technology can be utilized for mental health monitoring and early intervention. This could involve developing chatbots and mental health assessment systems to provide students with immediate emotional support and professional advice. (3) Longitudinal Research and Technological Integration: It is recommended to conduct longitudinal studies to assess the long-term impact of physical activity and AI-assisted tools on college students’ mental health. Such research will help understand the sustainability of intervention effects and guide future policy-making and practices.

## Data availability statement

The raw data supporting the conclusions of this article will be made available by the authors, without undue reservation.

## Ethics statement

The studies involving humans were approved by the Ethics Committee of the School of Basic Medical Sciences at Zhengzhou University. The studies were conducted in accordance with the local legislation and institutional requirements. The participants provided their written informed consent to participate in this study.

## Author contributions

SZ: Conceptualization, Data curation, Investigation, Methodology, Software, Supervision, Writing – original draft, Writing – review & editing, Formal analysis, Project administration, Validation, Funding acquisition, Resources, Visualization, Software. JC: Writing – original draft, Writing – review & editing, Data curation, Methodology, Supervision, Conceptualization, Formal analysis, Project administration, Validation, Investigation, Resources. YY: Writing – original draft, Data curation, Formal analysis, Funding acquisition, Investigation, Software, Supervision, Validation. HL: Funding acquisition, Resources, Supervision, Writing – review & editing. QZ: Funding acquisition, Resources, Supervision, Writing – review & editing. XW: Funding acquisition, Investigation, Resources, Supervision, Writing – review & editing.
